# The extent of lymph node yield in central neck dissection can be affected by preoperative and intraoperative assessment and alter the prognosis of papillary thyroid carcinoma

**DOI:** 10.1002/cam4.2762

**Published:** 2019-12-18

**Authors:** Jia‐Qian Hu, Duo Wen, Ben Ma, Ting‐Ting Zhang, Tian Liao, Xiao Shi, Yu‐Long Wang, Yong‐Xue Zhu, Yu Wang, Wen‐Jun Wei, Qing‐Hai Ji

**Affiliations:** ^1^ Department of Head and Neck Surgery Fudan University Shanghai Cancer Center Shanghai China; ^2^ Department of Oncology Shanghai Medical College Fudan University Shanghai China

**Keywords:** central neck dissection, lymph node yield, papillary thyroid cancer, surgical assessment

## Abstract

**Background:**

Lymph node yield (LNY) was implemented in the stratification of papillary thyroid cancer (PTC) patients. The effect of LNY may be related to the extent of surgery. This study aims to identify influencing factors for LNY in central compartment neck dissection (CND).

**Methods:**

Data of 13 712 consecutive PTC patients were analyzed retrospectively. Risk factors for LNY in CND and distribution characteristics of LNY were evaluated. Its relationship with prognosis was studied in another cohort of 136 cases.

**Results:**

LNY in therapeutic CND was significantly higher than prophylactic CND (Unilateral: 5.55 ± 3.79 vs 3.41 ± 2.77; Bilateral: 8.90 ± 5.10 vs 6.47 ± 4.17, *P* < .001). Other independent factors included extranodal extension (ETE), tumor size, and concurrent Hashimoto's thyroiditis. The inconsistency distribution of LNY in bilateral CND was associated with preoperative and intraoperative assessment. Patients with significant difference between major and minor LNY suffered from poorer prognosis (10y‐RFS: 58.3% vs 92.0%; HR = 6.719, 95%, *P* < .0001).

**Conclusions:**

CND surgical procedure, ETE, and Hashimoto's thyroiditis were independent factors of LNY. Inconsistent distribution of LNY was associated with prognosis of bilateral PTC patients. The impact of preoperative and intraoperative assessment on the actual extent of CND can be used to explain the relationship between LNY and PTC prognosis.

## Graphical abstract

Lymph node yield (LNY) in central compartment neck dissection (CND) of papillary thyroid cancer (PTC) is related to preoperative and intraoperative assessment of the disease. Inadequate LNY may lead to poor prognosis.

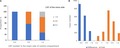

## 1 INTRODUCTION

Thyroid cancer is the most common neoplasm of endocrine system.^1^ Papillary thyroid cancer (PTC) accounts for more than 90% of all thyroid malignancies. Despite its inert biological behavior and good prognosis, lymph node metastasis in PTC is not rare, which is also a prognostic factor of recurrence.^2^ Complete resection of the primary tumor and regional lymph node metastases is regarded as the most critical treatment for PTC.

The specific features of lymph node metastases, including the number of positive lymph nodes (PLN), lymph node yield (LNY), lymph node ratio (LNR), and extranodal extension (ENE) have been suggested to serve as stratification parameters for patients with lymph node metastasis.^3^, ^4^, ^5^, ^6^, ^7^, ^8^, ^9^, ^10^, ^11^ PLN, LNR, and ENE all relates to tumor progression directly, consequently affecting PTC prognosis. However, this explanation is not applicable for LNY.

For differentiated thyroid cancer (DTC), the relationship between LNY and disease prognosis has already been well established.^4^, ^5^, ^12^, ^13^ Terry Hyslop et al^14^ presented a statistical model to estimate the risk of false‐negative lymph node dissection and occult nodal disease. LNY can reflect the actual extent of lymph node dissection. Inadequate extent of lymph node dissection may cause residual remnants of metastases, thus compromise patient prognosis.^15^

The real extent of dissection cannot be objectively evaluated, forcing us to turn to the indirect reflection of surgical extend, which is LNY. Based on the preoperative and intraoperative evaluation, a surgeon may form his own perception of the disease and adjust the actual extent of surgery. We aim to focus on LNY and its influencing factors in central compartment lymph node dissection (CND) in this study.

## 2 MATERIALS AND METHODS

### 2.1 Patients

Patients with histopathological diagnosis of PTC and received initial surgery at the Department of Head and Neck Surgery, Fudan University Shanghai Cancer Center, China, from January 2005 to December 2015 were recruited. The patients included in the study met the following criteria: (a) harboring no other type of malignant thyroid tumor; (b) no previous history of thyroid surgery; and (c) adequate medical history. All patients provided written informed consent for the use of their medical records.

### 2.2 Treatment

The management of PTC in our center was described previously.^16^ Briefly, all patients received an ultrasonography (US) examination preoperatively. Fine‐needle aspiration (FNA) was recommend for nodules ≥1 cm in greatest dimension with high suspicion sonographic patterns. Lobectomy plus ipsilateral CND was performed as the initial surgical treatment for patients with malignant lesions that were limited to a single lobe. Total thyroidectomy was performed when any of the following conditions is met: (a) bilateral malignant lesions; (b) gross extrathyroidal extension; and (c) distant metastasis. A modified lateral lymph node dissection (LLND), including levels II‐V, was performed only in cases with clinically evident lateral neck lymph node metastasis. All patients received TSH‐suppressive hormonal therapy after surgery. Radioactive iodine therapy was limited only to patients who had distant metastasis.

### 2.3 Clinicopathological data

Clinicopathological data (age, gender, maximum size of the largest lesion, ETE, multifocality, concurrent Hashimoto's thyroiditis, and lymph node metastasis) were collected. ETE of the primary tumor consisted of gross and microscopic ETE. Multifocal primary lesions were defined as two or more malignant lesions found within thyroid glands.

CND were divided into prophylactic and therapeutic CND. The judging criteria included: (a) whether or not suspicious lymph node was detected by preoperative US and (b) therapeutic CND was routinely performed in patients diagnosed with cN1b. We also defined a new term, “Semi‐therapeutic CND” in the study. It refers to CND performed in patients with inconclusive enlarged lymph nodes detected by preoperative US.

We further collected clinical data from an independent cohort consisting 136 patients with bilateral PTC and received bilateral CND during 2000‐2006 at our hospital. Prognosis information was all intact. A small portion of these patients overlaps the previous cohort. The recurrence‐free survival (RFS) was defined as the duration from the date of diagnosis by operation to the date of disease recurrence or progression. Patients with no event were censored on the date of the last follow‐up.

### 2.4 Statistics

The continuous data were expressed as mean ± standard deviation (SD). The statistical analysis was performed using a Student's *t* test or χ^2^ test, as appropriate. Multivariate analysis of association between clinicopathological features and LNY was performed using multiple linear regression. The Kaplan‐Meier method and log‐rank tests were employed to construct and compare survival curves. Multivariable analyses were performed by Cox proportional hazards regression models. Statistical analyses were performed using SPSS 22.0 (IBM Corp). Kaplan‐Meier survival curves were drawn by GraphPad Prism 6.01 (GraphPad Software). A two‐sided *P* value < .05 was considered as statistically significant.

## 3 RESULTS

### 3.1 Patient characteristics

The study cohort consisted of 13 712 patients, including 10 214 women with age range of 15‐88 years (mean 43.89 ± 11.87). Lobectomy or any other procedure less than total thyroidectomy was performed in 10 328 patients while 2733 patients underwent total thyroidectomy. CND was routinely performed in all patients. Therapeutic lateral lymph node dissection (LLND) was performed in 2285 patients. Incidence of positive lymph node metastasis in central and lateral compartment were 47.0% and 15.7%, respectively. The mean number of harvested central lymph nodes was 4.44 ± 3.71 (range 0‐37), and 26.14 ± 14.89 (range 5‐123) in lateral lymph nodes. The characteristics of these patients are listed in Table 1.

**Table 1:** Clinical and pathologic characteristics of 13 712 PTC patients

Clinical features	
Age at first diagnosis	
Mean (y)	43.89 ± 11.87
≤55 y	10 978 (80.1)
＞55 y	2734 (19.9)
Gender	
Male	3498 (25.5)
Female	10 214 (74.5)
Tumor size	
Mean (cm)	1.11 ± 0.86
≤10 mm	7278 (53.1)
＞10 mm	6434 (46.9)
ETE	
Negative	12 283 (89.6)
Minimal extension	1210 (8.8)
Advanced disease	219 (1.6)
Multifocal (%)	3876 (28.3)
Hashimoto thyroiditis (%)	2964 (21.6)
Surgery of primary tumor	
Lobectomy/less than total thyroidectomy	10 328 (75.3)
Total thyroidectomy	2733 (19.9)
Other	651 (4.7)
CND	
Therapeutic	2999 (21.9)
Prophylactic	10 713 (72.1)
Central neck lymph node metastasis (%)	6440 (47.0)
Lateral neck lymph node metastasis (%)	2149 (15.7)

Abbreviations: CND, central compartment lymph node dissection; ETE, extrathyroidal extension.

### 3.2 Impact of CND surgical procedure on number of LNY in central compartment

All patients were divided into three groups: (a) therapeutic CND group: patients diagnosed with cN1a by US and patients who needed LLND; (b) prophylactic CND group: patients who had no positive findings (cN0); and (c) semi‐prophylactic CND group: patients who were detected with indeterminate enlarged lymph nodes. As are listed in Table 2, regardless of unilateral or bilateral CND, patients with therapeutic CND had significantly higher LNY than prophylactic group. LNY of therapeutic group was also higher than semi‐prophylactic group, however not significantly.

**Table 2:** Influence of CND surgical procedure on the LNY in the central compartment

CND surgical procedure	Unilateral			Bilateral		
	N	LNY	*P* value	N	LNY	P value
Therapeutic	1915	5.55 ± 3.79	<.001	1084	8.90 ± 5.10	<.001
Semi‐therapeutic	647	4.98 ± 3.56		176	8.36 ± 4.39	
Prophylactic	8417	3.41 ± 2.77		1473	6.47 ± 4.17	

Abbreviations: CND, central compartment lymph node dissection; LNY, lymph node yield.

### 3.3 Impact of pathological features on LNY in CND

Univariate analysis in this study showed that ETE, tumor size, and concurrent Hashimoto's thyroiditis were associated with LNY in CND (Table 3). Although no difference of LNY between negative and minimal ETE group was detected, LNY in the gross ETE group was significantly higher than the above two (Unilateral：5.89 ± 3.76；Bilateral：8.90 ± 5.31, *P* < .001). Furthermore, multivariate regression analysis revealed that CND surgical procedure, ETE, and Hashimoto's thyroiditis were independent factors of LNY (Table 4).

**Table 3:** Influence of pathological features on LNY in central compartment

Clinical and pathological features	Unilateral			Bilateral		
	N	LNY	*P* value	N	LNY	*P* value
ETE			<.001			<.001
Negative	10 049	3.80 ± 3.01		2234	7.15 ± 4.64	
Minimal extension	822	3.67 ± 2.91		388	7.61 ± 4.0.71	
Gross extension	108	5.89 ± 3.76		111	8.90 ± 5.31	
Tumor size			.01			.012
≤10 mm	7157	3.57 ± 2.89		1328	7.07 ± 4.46	
＞10 mm	3822	4.26 ± 3.36		1405	8.10 ± 4.93	
Hashimoto thyroiditis			<.001			<.001
Negative	8761	3.41 ± 2.81		1987	6.75 ± 4.33	
Positive	2218	5.37 ± 3.55		746	9.60 ± 5.04	
Multifocality			.112			‐
Negative	9476	3.79 ± 3.07		‐	‐	
Positive	1503	3.93 ± 3.13		‐	‐	

Abbreviations: ETE, extrathyroidal extension; LNY, lymph node yield.

**Table 4:** Multivariate analysis of association between clinical/pathological features and LNY in central compartment

Clinical/pathological features	Unilateral		Bilateral	
	Coefficients	*P* value	Coefficients	*P* value
CND surgical procedure	0.264	<.001	0.224	<.001
ETE	0.035	.01	0.059	.01
Tumor size	0.01	.291	0.033	.078
Hashimoto thyroiditis	0.247	<.001	0.293	<.001

Abbreviations: CND, central compartment lymph node dissection; ETE, extrathyroidal extension.

### 3.4 Characteristics of LNY in patients with bilateral CND

We identified 2246 patients underwent total thyroidectomy plus bilateral CND. For the convenience of this analysis, the side with more LNY was defined as the major side, and the less as the minor side. Accordingly, 456 patients had a major LNY less than 2 while their minor LNY was mostly 0. These patients were excluded considering difficulty of following analysis. Eventually, 1790 patients entered the analysis phase.

**Figure 1:**
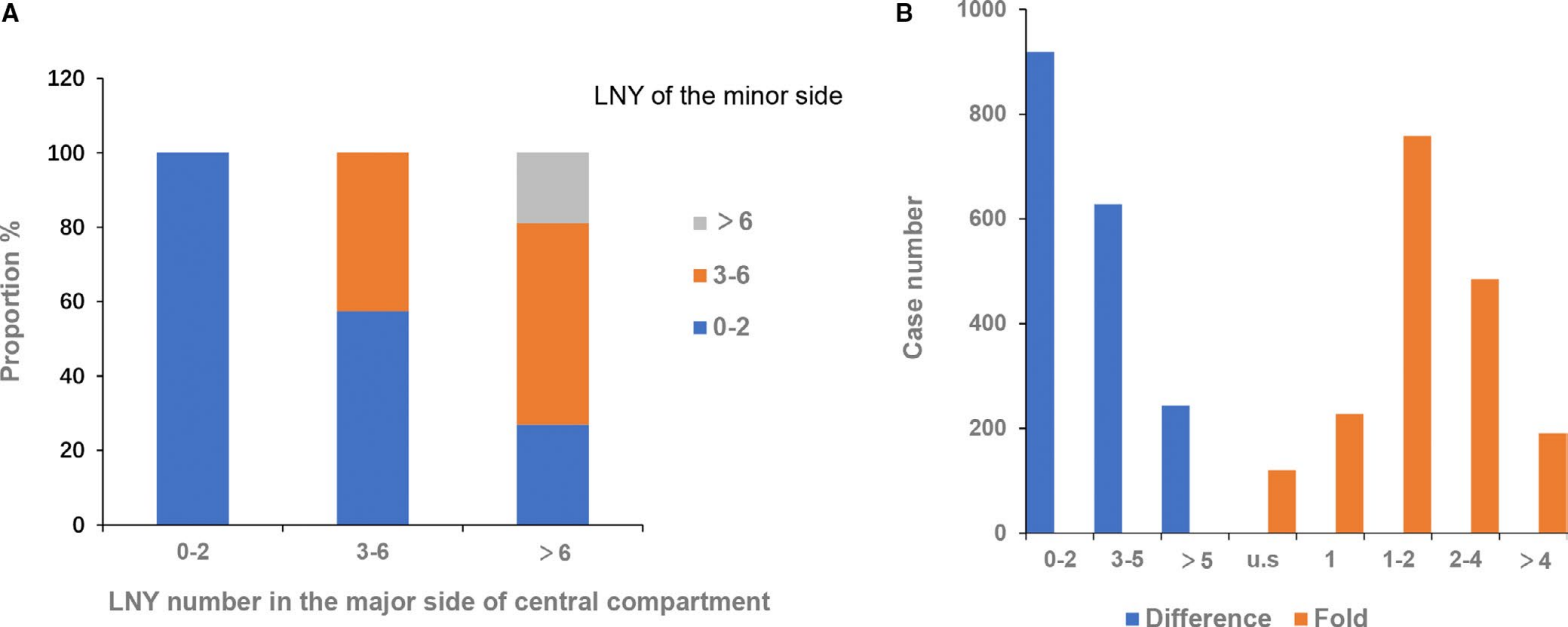
Difference and ratio relationships of LNY between major and minor side. A, Detailed paired information of LNY in both sides. B, Patient distribution based on the difference and ratio value of LNY between major and minor sides. US: ratio value cannot be calculated because the LNY of minor side is 0. Difference: LNY (major‐minor). Ration: LNY (major/minor). 456 cases with LNY ≤2 in major side were excluded for ratio calculation

Difference and ratio relationships of LNY between the major and minor side are presented in Figure 1. When the major LNY was more than 6, only 18.9% of the patients has the same number of minor LNY. In 48.7% and 13.6% of the patients, the difference between major and minor LNY was ≥3 and ≥6, respectively. In terms of LNY ratio, 38.3% of the patients had a major LNY which was 2 times higher than the minor LNY, and 10.6% had a LNY ratio of more than 4. The proportion would rise to 41.0% and 11.4%, respectively, if 120 patients with minor LNY of 0 were excluded (Table S1).

Patients were then divided into three groups: (a) bilateral prophylactic CND; (b) unilateral therapeutic and contralateral prophylactic CND; and (c) bilateral therapeutic CND. In group 3, patients whose LNY difference were less than 2 and greater than 6 accounted for 58.6% and 9.7% of the total population, respectively. Similar data can be seen in group 1 (56.7% and 11.0%). However, it was significantly different in group 2, with the corresponding data of 33.9% and 22.3%, respectively. Similar results were also observed in the ratio analysis (Table 5). Bilateral LNY in patients of group 2 had the greatest degree of variation.

**Table 5:** Relationship between surgical procedure and difference of LNY in PTC patients underwent bilateral central neck dissection

	Surgical procedure of CND			*P* value
	Bilateral prophylactic CND	Prophylactic + Therapeutic CND	Bilateral Therapeutic CND	
Difference				
≤2	562 (56.7)	152 (33.9)	205 (58.6)	<.001
2‐6	321 (32.3)	196 (43.8)	111 (31.7)	
≥6	109 (11.0)	100 (22.3)	34 (9.7)	
Ratio				
1	160 (16.1)	25 (5.6)	42 (12.0)	
1‐2	377 (38.0)	170 (37.9)	211 (60.3)	
2‐4	267 (26.9)	158 (35.3)	70 (20.0)	
>4	105 (10.6)	63 (14.1)	22 (6.3)	
Uncalculated^a^	83 (8.4)	32 (7.1)	5 (1.4)	

Abbreviations: CND, central compartment lymph node dissection; LNY, lymph node yield.

a Uncalculated: ratio value cannot be calculated because the LNY of minor side is 0. Difference: LNY (major‐minor). Ration: LNY (major/minor). 456 cases with LNY ≤2 in major side were excluded for ratio calculation.

### 3.5 Impact of LNY pattern in patients with bilateral CND on survival

In a considerable portion of patients with bilateral CND, the differences in LNY between the major and minor side was significant. Data from 136 bilateral PTC patients with bilateral CND during 2000 to 2006 were collected. The average follow‐up period was 107 months (range from 8 to 201 months). Detailed information of these cases is listed in Table S2. At the endpoint of our follow‐up, 24 patients suffered from recurrent disease. Among them, 11 cases had recurrent sites in the lateral neck (level II‐V), 7 cases in the level VI and thyroid bed, 4 cases in both of lateral and central compartment and 2 cases with distant metastasis.

**Figure 2:**
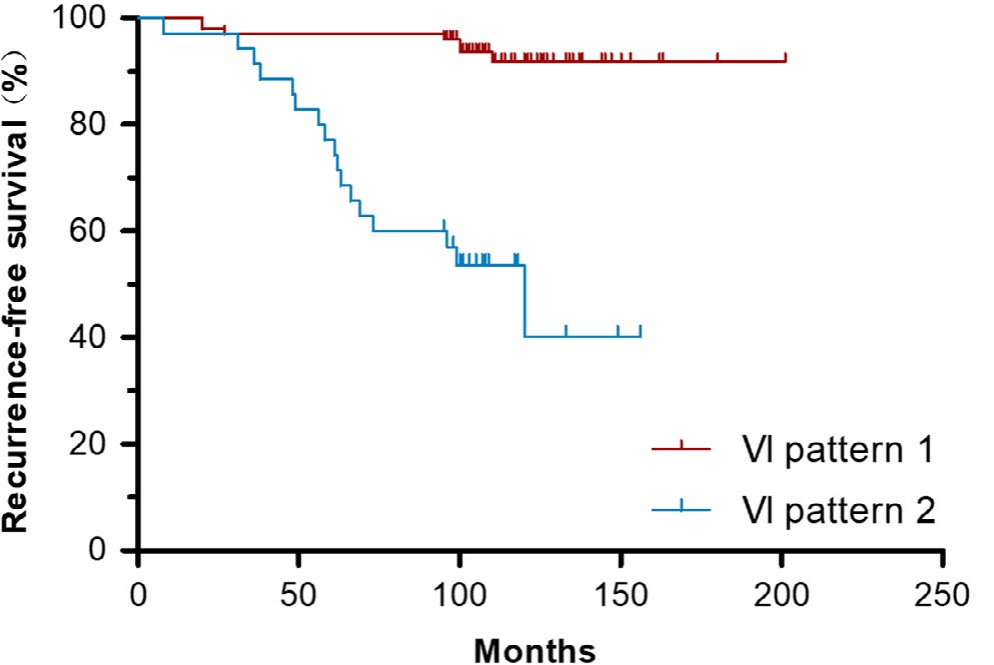
Kaplan‐Meier plots of recurrence‐free survival. The survival plot grouped by VI pattern indicated a significant difference of RFS between patients with different distributional characteristics of bilateral LNY (*P* = .001). VI pattern 1: non‐significant group with D‐value ≤3 or R‐value <2; VI pattern 2: significant group with D‐value >3 and R‐value ≥2

We then divided all cases into two groups according to LNY disparity: (1) non‐significant group with difference value (D‐value) ≤3 or ratio value (R‐value) <2 and (2) significant group with D‐value >3 and R‐value ≥2. In the 24 relapsed patients, 17 (70.8%) were classified as significant group. From Kaplan‐Meier survival plots (Figure 2), we observed a significant worse prognosis in this group. The calculated 5y and 10y‐RFS was 96.0% and 92.0% for non‐significant group, as well as 80.6% and 58.3% for significant group. This result was also supported by Cox regression analysis after adjusted by age, gender, T stage, and local metastatic status (HR = 6.719, 95% CI: 2.789‐16.789, *P* < .0001), indicating LNY distribution of CND is an independent factor of RFS in bilateral PTC patients.

Recurrence characteristics of the 17 relapsed cases are summarized in Table 6. Of these 17 cases, the central neck compartment was involved in 10 cases (58.8%) and the shortest RFS time was 31m, indicating a direct relationship between the extent of CND on the minor side and disease recurrence in PTC patients.

**Table 6:** Clinical characteristics of 17 recurrence cases with significantly diverse LNY in major and minor side

Case	Age	Gender	Major side (PLN/LNY)	Minor side (PLN/LNY)	Location of recurrence lesion	Recurrence‐free survival (m)
1	24	Female	9/9	3/3	Level VI in major side Lateral neck in minor side	8
2	51	Male	4/6	0/0	Lateral neck in major side	96
3	49	Male	8/11	3/3	Lateral neck in minor side	48
4	33	Female	12/16	1/7	Level VI and lateral neck in minor side	120
5	24	Male	12/13	0/0	Level VI and lateral neck in minor side	49
6	35	Male	3/4	0/0	Level VI in minor side	73
7	42	Female	5/8	2/2	Level VI in both sides	58
8	15	Male	7/7	3/3	Level VI in both sides	63
9	27	Female	4/14	2/4	Lateral neck in major side	61
10	28	Female	13/15	5/5	Lateral neck in major side	66
11	37	Female	11/13	3/3	Level VI and lateral neck in minor side	31
12	46	Male	7/14	6/8	Level VI in both sides	36
13	55	Female	3/8	1/3	Level VI in both sides and thyroid cartilage	62
14	52	Female	3/14	0/1	Lateral neck in major side	69
15	22	Female	6/9	2/4	Level VI in minor side	56
16	50	Female	3/5	1/1	Lung and subcutaneous metastasis	38
17	59	Female	7/7	2/2	Mass in central neck with trachea, larynx, prevertebral invasions	99

Abbreviations: LNY, lymph node yield; PLN, positive lymph node.

## 4 DISCUSSION

The relationship between LNY and the prognosis of PTC has been well established by previous studies.^4^, ^5^, ^12^, ^13^ Higher LNY is associated with a lower recurrent rate, regardless of CND or LND. The risk factors of LNY could be summarized into three categories: (a) individual factor. Anatomic difference between individuals. According to Tavares MR et al,^17^ the normal number of lymph nodes in the central neck compartment varies from 2 to 44. (b) Pathological factor. Under the circumstance of small lymph nodes and relatively large dissection specimen, lymph nodes may be hard to distinguish and thus cloud the count of LNY.^18^ (c) Surgical factor. LNY is an indirect reflection of the extent of neck dissection. Therefore, surgical factor has the highest correlation with clinical practice.

Difference in LNY can be interpreted in two ways, the CND was either expanded or narrowed. It is reasonable to assume that some surgeons would expand the scope of CND in order to achieve the best curative effect. Theoretically, an enlarged CND should lead to the same prognosis as that of a standard CND. However, a significantly higher risk of recurrence was noticed in imbalanced bilateral CND patients in our study, indicating the difference of LNY was mainly caused by the narrowing of CND.

We have analyzed several suspicious factors that may alter a surgeon's perception of the disease, causing variation in the completeness of CND and further affect LNY. According to our data, we have reached the following important conclusions: (a) Preoperative factors. When suspicious lymph nodes were detected by US (cN1), LNY obtained by therapeutic CND was significantly higher than prophylactic surgery. (b) Intraoperative factors. Among all analyzed pathological characteristics, gross ETE and Hashimoto's thyroiditis were the only two independent factors. (c) For patients undergoing bilateral CND, it was common to observe LNY disparity on both sides. Notably, patients with greater LNY difference had a significantly higher risk of recurrence.

The actual extent of CND, as reflected by LNY, was varied due to the purpose of CND. The appearance of the tumor and lymph nodes presented during operation are more intuitive. Our study showed in patients with minimal ETE, LNY was almost the same as the patients without any ETE. However, significantly increased LNY was detected when gross ETE occurred. Tumors with gross ETE were more easily observed by the naked eye, causing surgeons to expand CND extent, which can also be applied in Hashimoto's thyroiditis where hyperplastic lymph nodes appeared. However, the long‐term inflammation itself may increase the number of regional lymph node.^19^, ^20^

We then tried to perform a similar analysis using SEER database. As SEER did not separate CND from other kinds of neck dissections, we could only include patients with staging N0 and N1a. (N = 8974). CND surgical procedure, Hashimoto's thyroiditis, and any other related characteristics were insufficient in the SEER database. Therefore, we only conducted a univariate analysis of ETE and tumor size. As listed in Table S3, both factors were significantly associated with LNY.

The results of bilateral CND and LNY should be interpreted with caution. The quantity of lymph nodes in the left and right side of central neck compartment is anatomically different. Also, the borderline which separate the left and right central compartment is simply a man‐made cut. Therefore, the variation of LNY caused by a surgeon's distribution of the lymph nodes was treated as a random factor. Similarly, negligence in pathologic reports was considered as another random factor. All the mentioned factors should have no effect on prognosis in theory. However, our results showed that prophylactic plus therapeutic CND caused an increased difference of LNY between two sides and was accompanied by a significant reduction in RFS, indicating a true divergence in patient prognosis and emphasizing the influence of the surgical extent. CND is a known risk factor for injuries of recurrent laryngeal nerve and parathyroid gland. Upon that, bilateral CND may lead to permanent tracheotomy and hypocalcemia.^21^ To avoid complications, the actual extent of CND on the prophylactic side may be reduced if no other risk factors were observed.

Our study has some limitations. First, the routine surgical approach in our hospital is different from most of other centers in the world. Second, for 136 bilateral PTC patients with prognostic analysis, information of RAI was insufficient, which may affect the related results. Third, because of limited follow‐up data, we did not analyze the relationship between LNY and prognosis in the entire patient population. Fourth, it was a single‐institution retrospective study, results of which need to be confirmed by more evidence in the future.

In conclusion, by analyzing the data from a relatively large patient sample, we found that the CND procedure, ETE of primary tumor, and concurrent Hashimoto's thyroiditis were independent factors of LNY. In PTC patients with bilateral CND, LNY in one side was often higher than that of the other. This phenomenon was also associated with the CND procedure and accompanied with a significantly increased risk of recurrence. These results suggest that preoperative and intraoperative assessment suggesting the patient is at higher risk impacts on the actual extent of CND, which may explain the relationship between LNY and the prognosis of PTC patients.

## CONFLICT OF INTEREST

No competing financial interests exist.

## Data Availability

The data in this article can be available via corresponding author.
