# Supplementary material for: The extent of lymph node yield in central neck dissection can be affected by preoperative and intraoperative assessment and alter the prognosis of papillary thyroid carcinoma
Source: Cancer Med. 2019 Dec 18;9(3):1017–24. doi: 10.1002/cam4.2762 (PMC6997063; doi:10.1002/cam4.2762)
Supplement: Supplementary file 1 [file CAM4-9-1017-s001.docx]

**Supplemental table 1 Detailed information of LNY in major and minor side**

| LNY of major side | LNY of minor side | | |
| --- | --- | --- | --- |
|  | 0-2 | 3-6 | >6 |
| 0-2 | 456 (100.0) | 0 (0.0) | 0 (0.0) |
| 3-6 | 688 (57.4) | 510 (42.6) | 0 (0.0) |
| >6 | 159 (26.9) | 321 (54.2) | 112 (18.9) |

|  | Value | Frequency |
| --- | --- | --- |
| LNY Difference (major-minor) | | |
| 0-2 | 919 | 51.3 |
| 3-5 | 628 | 35.1 |
| ≥6 | 243 | 13.6 |
| LNY Ratio (major/minor) | | |
| 1 | 227 | 12.7 |
| 1-2 | 758 | 42.3 |
| 2-4 | 495 | 27.7 |
| >4 | 190 | 10.6 |
| Uncalculated^*^ | 120 | 6.7 |

^*^Uncalculated: ratio value cannot be calculated because the LNY of minor side is 0.

456 cases with LNY ≤2 in major side were excluded.

**Supplemental Table 2** **Clinical and pathologic characteristics of 136 bilateral PTC patients received bilateral CND**

| **Clinical features** | |
| --- | --- |
| **Age at first diagnosis** | |
| Mean (y) | 42.71±13.50 |
| ≤55y | 112 (82.4) |
| ＞55y | 24 (17.6) |
| **Gender** | |
| Male | 59 (43.4) |
| Female | 77 (56.6) |
| **Tumor size** | |
| Mean (cm) | 1.11±0.86 |
| ≤10mm | 41 (30.1) |
| ＞10mm | 95 (69.9) |
| **ETE** | |
| Negative | 84 (61.8) |
| Minimal extension | 39 (28.7) |
| Advanced disease | 13 (9.6) |
| **Central neck lymph node metastasis (%)** | 116 (85.3) |
| **Lateral neck lymph node metastasis (%)** | 52 (38.2) |
| **Follow-up** | |
| Mean (months) | 107 |
| Range | 8-207 |

CND: central compartment lymph node dissection; ETE: extrathyroidal extension.

**Supplemental table 3 Influence of tumor size and ETE on the LNY in central compartment: data from SEER database**

|  | **Unilateral** | | |
| --- | --- | --- | --- |
|  | **N** | **LNY (mean±SD)** | **P value** |
| ETE |  |  | 0.018 |
| Negative or minimally invasive ( T1-3) | 8765 | 4.47±6.13 |  |
| Advanced disease (T4) | 153 | 6.05±8.09 |  |
| unknown | 56 | - |  |
| Tumor Size |  |  | <0.001 |
| ≤1cm | 3590 | 3.83±5.74 |  |
| 1-2cm | 2608 | 4.77±5.74 |  |
| 2-4cm | 1955 | 5.21±6.85 |  |
| >4cm | 691 | 4.93±7.24 |  |
| unknown | 130 | - |  |
